# *Salmonella* chitosan nanoparticle vaccine administration is protective against *Salmonella* Enteritidis in broiler birds

**DOI:** 10.1371/journal.pone.0259334

**Published:** 2021-11-16

**Authors:** Keila Y. Acevedo-Villanueva, Sankar Renu, Revathi Shanmugasundaram, Gabriel O. Akerele, Renukaradhy J. Gourapura, Ramesh K. Selvaraj

**Affiliations:** 1 Department of Poultry Science, The University of Georgia, Athens, Georgia, United States of America; 2 Department of Veterinary Preventative Medicine, Center for Food Animal Health, Ohio Agricultural Research and Development Center, The Ohio State University, Columbus, Ohio, United States of America; 3 USDA-ARS, Toxicology and Mycotoxins Research Unit, Athens, Georgia, United States of America; Tokat Gaziosmanpasa Universitesi, TURKEY

## Abstract

*Salmonella* control strategies include vaccines that help reduce the spread of *Salmonella* in poultry flocks. In this study we evaluated the efficacy of administering a live *Salmonella* vaccine followed by a killed *Salmonella* chitosan nanoparticle (CNP) vaccine booster on the cellular and humoral immunity of broilers. The CNP vaccine was synthesized with *Salmonella* Enteritidis (*S*. Enteritidis) outer-membrane-proteins (OMPs) and flagellin-proteins. At d1-of-age, one-hundred-sixty-eight chicks were allocated into treatments: 1) No vaccine, 2) Live vaccine (Poulvac^®^ST), 3) CNP vaccine, 4) Live+CNP vaccine. At d1-of-age, birds were orally vaccinated with PBS, Live vaccine, or CNP. At d7-of-age, the No vaccine, Live vaccine and CNP vaccine groups were boosted with PBS and the Live+CNP vaccine group was boosted with CNP. At d14-of-age, birds were challenged with 1×10^9^ CFU/bird *S*. Enteritidis. There were no significant differences in body-weight-gain (BWG) or feed-conversion-ratio (FCR). At 8h-post-challenge, CNP and Live+CNP-vaccinated birds had 17% and 24% greater levels (P<0.05) of anti-*Salmonella* OMPs IgA in bile, respectively, compared to control. At d28-of-age, CNP, Live, and Live+CNP-vaccinated birds had 33%, 18%, and 24% greater levels (P<0.05) of anti-*Salmonella* OMPs IgA in bile, respectively, compared to control. At d14-of-age, Live+CNP-vaccinated birds had 46% greater levels (P<0.05) of anti-*Salmonella* OMPs IgY in serum, compared to control. At d21-of-age, splenocytes from CNP and Live-vaccinated birds had increased (P<0.05) T-lymphocyte proliferation at 0.02 mg/mL OMPs stimulation compared to the control. At d28-of-age, CNP and Live+CNP-vaccinated birds had 0.9 Log10 CFU/g and 1 Log10 CFU/g decreased *S*. Enteritidis cecal loads (P<0.05), respectively, compared to control. The CNP vaccine does not have adverse effects on bird’s BWG and FCR or IL-1β, IL-10, IFN-γ, or iNOS mRNA expression levels. It can be concluded that the CNP vaccine, as a first dose or as a booster vaccination, is an alternative vaccine candidate against *S*. Enteritidis in broilers.

## Introduction

*Salmonella* is a major known zoonotic, foodborne pathogen that signifies a public health concern in the United States of America (USA). The Centers for Disease Control and Prevention (CDC) estimated that in the USA approximately more than one million foodborne salmonellosis cases occur annually [1]. In the USA, approximately more than 70% of human salmonellosis cases have been linked to the consumption of contaminated chicken or eggs [2]. Non-typhoidal *Salmonella* serovars, like *Salmonella* enterica serovar Enteritidis (*S*. Enteritidis), survives the acidic pH of the gastrointestinal tract, invades the intestinal mucosal cells and the underlying lymphoid tissues, and proliferates. Once *Salmonella* invades the host it causes salmonellosis, which can be manifested as diarrhea, fever, and abdominal pain. Unlike humans, chickens can tolerate high loads of *Salmonella*, up to Log 5 CFU [3], and remain asymptomatic. However, poultry is one of the main reservoirs for *Salmonella*, making poultry meat and eggs a risk source for the spread of this pathogen. With the increasing population and industrialization of poultry and poultry products, effective *Salmonella* control strategies are crucial.

*Salmonella* killed vaccines for poultry are preferred over the use of live vaccines due to the potential risks that *Salmonella* live vaccines can cause. Live vaccines can revert their virulence [4], which increases the risk of environmental contamination and compromises the flock’s health. Killed vaccines are known to induce a “weaker” immune response, as they elicit a lower cell-mediated immunity, while live vaccines elicit both cell-mediated and antibody-mediated immune responses [5]. However, killed vaccines do not pose any risk of reverting the bacterial strain pathogenicity. Nonetheless, *Salmonella* killed vaccines for broilers are administered individually by subcutaneous injection [6]. Individual subcutaneous injections are impractical when handling commercial poultry flocks, but moreover, if they are not done properly it can result in focal inflammatory myositis [7] and can decrease the quality of the tissue.

In the poultry industry, mass-vaccination is a key to maximize the protection of big poultry flocks in a more efficient and faster way while decreasing labor costs. The delivery of vaccine antigens using the oral route allows for mass vaccination benefits while inducing mucosal immunity [8], which is essential for poultry because most disease-causing bacteria colonize the gastrointestinal tract. However, there is currently no commercially available oral killed vaccine against *Salmonella* for use in poultry. The acidic environment of the gastrointestinal tract challenges the delivery of unprotected vaccine antigens to the target intestinal tissues [9]. An oral killed vaccine for broilers against *Salmonella* eliminates the risk of the bacterial strain reverting to its virulent form, increases the bird’s welfare and mucosal immune response, and reduces the labor cost.

*Salmonella* poultry vaccines have a withdrawal period of twenty-one days before slaughter. Live vaccines require two doses, three to four weeks apart, whereas inactivated vaccines can be administered simultaneously or after live vaccines [10]. For an optimal level of protection without severe adverse reactions, vaccination programs can include the sequential use of live-attenuated vaccines followed by inactivated vaccines [11, 12]. Killed vaccines can induce high and uniform levels of protecting antibodies after administrating a live vaccine [12, 13], which helps sustain the immune response against the organism of interest. The administration of a live vaccine followed by a killed vaccine against *Salmonella* can provide longer-lasting protection [12], which could in the long-term reduce the producers’ cost by decreasing the chances of *Salmonella* outbreaks in their facilities. Administering a live vaccine followed by a killed vaccine booster can eliminate the time restrictions of live vaccine boosters and allow producers to comply with the withdrawal period.

Recently, *Salmonella* polymeric nanoparticle vaccines for poultry have been characterized and used for antigen delivery to the gastrointestinal tract [9, 14, 15]. Our novel chitosan and polyanhydride nanoparticle vaccines against *Salmonella* were synthesized with crude-enriched outer membrane proteins (OMPs) and flagellin protein extracts from *S*. Enteritidis and surface-tagged with flagellin proteins [9, 16]. Findings from our previous studies with both chitosan and polyanhydride nanoparticle vaccines against *Salmonella* have shown that: it can be administered orally, aid in target delivery of the antigen to the mucosal Peyer’s Patches, are biocompatible with chickens, have no adverse effect on bird’s performance or health, can substantially increase an antigen-specific mucosal immune response, and can decrease the *Salmonella* load in the ceca [9, 14, 16, 17]. More specifically, the chitosan nanoparticle (CNP) vaccine under study has shown to possess a high cationic charge and spherical shape with an average particle size distribution of approximately 500 nm, with an encapsulation efficacy for *Salmonella* antigens of 70% and a 40% efficacy of surface conjugation of flagellin protein, and no hemolysis of red blood cells upon treatment with CNPs [9].

This study evaluated the efficacy of administering a live *Salmonella* vaccine followed by a killed *Salmonella* CNP vaccine booster on broilers. We hypothesize that the CNP vaccination and CNP booster vaccination can induce an antigen-specific immune response against *S*. Enteritidis and significantly decreases *S*. Enteritidis cecal load on broilers. We tested our hypothesis by 1) quantifying the serum, cloacal swabs, and bile anti-*Salmonella* OMPs IgY and IgA antibodies, 2) quantifying the *S*. Enteritidis loads in ceca, 3) quantifying the antigen-recall response, 4) quantifying the IL-1β, IL-10, IFN-γ, and iNOS mRNA amounts, and 5) monitoring the weekly body-weight-gain (BWG) and feed-conversion-ratio (FCR) of vaccinated and experimentally challenged broilers.

## Materials and methods

### Ethical considerations

All animal protocols were approved by the Institutional Animal Care and Use Committee (IACUC) at the University of Georgia. The ethical approval number is A2018 03-021-Y3-A6. Research and all experimental procedures were performed following the pertinent guidelines concerning animal handling and animal care and welfare.

### Experimental animals

Broiler birds (Cobb-Vantress hatchery, Inc. Cleveland, GA, USA) had access to *ad libitum* feed and water. Birds were monitored at least once a day for dehydration, refusal to eat food, loss of body weight, diarrhea, bloody feces, and lethargy during the experimental period. All birds were euthanized at different time points and at the end of the experimental period with CO_2_, as per the IACUC standards.

#### Treatment groups

At d1 of age, one hundred sixty-eight chicks were randomly allocated into four treatment groups: 1) No vaccine, 2) Live vaccine, 3) CNP vaccine, 4) Live+CNP vaccine. There is currently no commercially available oral killed vaccine against *Salmonella* for use in broilers; hence, as a positive control a *Salmonella* live vaccine for broilers was used in this study. The *Salmonella* live vaccine used for this study was POULVAC^®^ ST (Zoetis, USA), prepared as per manufacturer’s instructions for delivery in the drinking water. At day of vaccination, all treatments were delivered by oral gavage. The mock PBS (7.4 pH) was given in a volume of 1 mL per bird, the CNP was given at 10 μg per bird in 1 mL PBS, and the Live vaccine was delivered in 1 mL of distilled water. For this experiment, the experimental unit was the pen, n = 6 pen/treatment. After vaccination, each pen was randomly assigned 7 technical replicates as birds/pen. A summary of the treatment groups is provided in Table 1.

**Table 1:** Experimental design showing assignment of broiler birds’ groups^1^.

Group	Experimental Treatment	Challenge
No vaccine	Mock PBS	*Salmonella* Enteritidis
Live vaccine	POULVAC^®^ ST vaccine	
CNP vaccine	10μg killed CNP vaccine	
Live+CNP vaccine	POULVAC^®^ ST vaccine + 10μg killed CNP vaccine booster	

^1^For all experimental groups, the experimental unit was the pen, n = 6 pen/treatment, with 7 technical replicates as birds/pen.

At day of vaccination, all treatments were delivered by oral gavage. The mock PBS (7.4 pH) was given in a volume of 1 mL per bird, the CNP was given at 10 μg per bird in 1 mL PBS, and the Live vaccine was delivered in 1 mL of distilled water.

### Synthesis of chitosan nanoparticle vaccine

#### Isolation of outer membrane proteins

A pure culture of wild-type *Salmonella* Enteritidis was used for the experimental challenge. *Salmonella* Enteritidis was grown in Tryptic Soy Broth (G-Biosciences, MO) for 48 hours at 37°C with shaking. The cells pellet were collected after washing two times with Phosphate-Buffered Saline (PBS) (pH 7.4) by centrifugation at 4,800xg for 40 minutes (Beckman Coulter, Avanti J15R). The OMPs were isolated as described previously [9] with a few modifications. The enriched bacterial cell pellet was collected and washed two times using 10 mM TRIS Base buffer (pH 7.5). The bacterial cell pellet was heat-killed at 75°C for 10 minutes. The cell pellet was treated with 2% Triton X-100 in 10 mM Tris HCl buffer (pH 7.5) and the killed bacterial cells were disrupted using a laboratory homogenizer (OMNI Inc., GA) for two rounds of 3 minutes each; allowing for a small cooling period in between. The cell suspension was centrifuged at 4,800 ×g for 30 minutes. The supernatant was collected and ultra-centrifuged (Beckman Coulter Optima^™^ L-90K) at 100,000 ×g for 3 hours. The protein concentration was estimated as per the manufacturer’s instructions, using Bradford Protein Assay (Bio-Rad, USA). The pellet containing OMPs-enriched extract was freeze-dried with 5% sucrose as a cryoprotectant.

#### Isolation of flagellar proteins

The flagellin proteins were isolated as described previously [9] with a few modifications. In brief, *S*. Enteritidis stock culture was inoculated into Brain Heart Infusion Broth (Sigma-Aldrich, MO) and grown for 48 hours at 37°C without shaking. The bacterium was washed two times with PBS (pH 7.4) by centrifugation at 4,000 ×g for 40 minutes. The bacterial cell pellet was treated with 3M Potassium Thiocyanate in PBS for 2 hours at room temperature with magnetic stirring. The cell suspension was ultracentrifuged at 35,000 ×g for 30 minutes. The supernatant containing the flagellin protein-enriched extract was dialyzed against PBS (pH 7.4) followed by dialysis in Milli-Q water. The protein concentration was assessed and freeze-dried using 5% sucrose as a cryoprotectant.

#### Preparation of loaded chitosan nanoparticle vaccine

The CNP vaccine was synthesized at the Ohio Agricultural Research and Development Center, The Ohio State University, USA. The OMPs and flagellin proteins isolated from *S*. Enteritidis were used to synthesize the vaccine using the ionic gelation method, as described previously [9]. In brief, a solution of 1.0% (w/v) low molecular weight chitosan (Sigma-Aldrich, MO) was prepared by slowly dissolving chitosan in an aqueous solution of 4.0% acetic acid. Afterwards, the solution was sonicated, and the pH was adjusted to 4.3. The solution was filtered using a 0.44 μm syringe filter. Five milliliters of the 1.0% chitosan solution was added to 5 mL of dH2O and incubated with 2.5 mg OMPs and flagellar proteins. To form the nanoparticles, 2.5 mL of 1% (w/v) Sodium Tripolyphosphate (TPP) in 2.5 mL deionized water was added to the above solution under magnetic stirring at room temperature. Then, to surface-conjugate the nanoparticles with flagellin proteins, 2.5 mg of flagellin protein in PBS were added to the nanoparticles. The CNP vaccines were collected by centrifugation at 10,500 ×g for 10 minutes, lyophilized, and stored at -80 °C.

### Sample collection and preparation

For this experiment, the experimental unit was the pen, n = 6 pen/treatment, with 7 technical replicates as birds/pen. At day of hatch, chicks were screened for *Salmonella* by enrichment of cloacal swab samples in Tetrathionate Broth (Neogen, MI) for 6 h followed by the inoculation of 10 μl of the enriched-supernatant to Modified Semi-Solid Rappaport-Vassiliadis (MSRV) Agar (Neogen, MI). At d1 of age, forty-two birds/group were orally vaccinated with either mock PBS, POULVAC^®^ ST vaccine, or CNP vaccine. At d7 of age, birds in the No vaccine, Live vaccine and CNP vaccine groups were boosted with mock PBS and birds in the Live+CNP vaccine group were boosted with CNP vaccine. At d14 of age, all birds were orally challenged with 1×10^9^ CFU/bird *S*. Enteritidis. At d1, d7, d14, and d21 of age, body weight and feed consumption were recorded. Body weight gain (BWG) and feed consumption ratio (FCR) were calculated. Samples were collected from one bird/pen (n = 6) at each time point. Blood and cloacal swabs were collected at d7, d14, 8h post-challenge, d21 and d28 of age. Serum and cloacal swabs were analyzed by enzyme-linked immunosorbent assay (ELISA) for anti-OMPs specific IgY and IgA antibodies, respectively. At 8h-post-challenge, d21, and d28 of age, anti-*Salmonella* OMPs IgA in bile, were analyzed. At d28 of age, the birds’ ceca were collected for *S*. Enteritidis quantification by plating. At d28 of age, primary splenocytes were isolated and pulsed with *Salmonella* OMPs or flagellin to determine the recall response, and cecal tonsils were collected to analyze inflammatory cytokines IL-1β and IFN-γ, anti-inflammatory cytokine IL-10, and induced nitric oxide synthase (iNOS) mRNA levels by RT-PCR.

#### Anti-OMPs specific IgY and IgA antibodies in serum, cloacal swabs, and bile of vaccinated birds

Serum, cloacal swabs, and bile were collected from one bird/pen (n = 6) at each time point. The amounts of antigen-specific antibodies in serum, cloacal swab, and bile samples were determined by ELISA, as described previously [9]. All treatment groups consisted of six samples, in duplicates, for each time point. High-binding-flat bottom 96-well plates (ThermoFisher Scientific, MA) were coated with OMPs, 2μg/mL for IgG and 7.5 μg/mL for IgA, diluted in 0.05 M sodium -bicarbonate coating buffer (9.6 pH). Plates were incubated overnight at 4°C no shaking. Plates were washed three times with PBS- Tween 20 (PBST) (0.05% Tween 20) (pH 7.4) and blocked with 5% non-fat dry milk powder in PBST for 1 hour at 37°C. Plates were washed three times with PBST. Fifty microliters of serum (1:10) and bile (1:200) samples were diluted in 2.5% non-fat dry milk, and 50 μl of undiluted cloacal supernatants were added to each well. Samples were incubated for 2 hours at 37°C and were washed three times with PBST. Fifty microliters per well of HRP-conjugated goat anti-chicken IgG (Cat. No. 6100–05, Southern Biotech, AL) (1: 10,000 in 2.5% non-fat dry milk powder in PBST) or HRP-conjugated goat anti-chicken IgA (Cat. No. A30-103P, Bethyl Laboratories, TX) (1: 3000 in 2.5% skim milk powder in PBST) secondary antibodies were added and incubated for 2 hours at 37°C. Plates were washed three times with PBST, and 50 μl of TMB peroxidase substrate (1:1 mixture of TMB peroxidase substrate and peroxidase substrate solution B) (KPL,MD) was added to each well. The reaction was stopped after 6 min by adding 2M Sulfuric Acid (J.T. Baker Inc., NJ). The Optical Density (OD) was measured at 450 nm using a spectrophotometer (BioChek, USA). The corrected OD was obtained by subtracting the treatment group OD from the blank control OD. The IgG and IgA values were reported as the mean optical density.

#### *Ex-vivo* recall-response of splenic PBMCs of vaccinated birds

Spleen samples were collected from one bird/pen (n = 6) at d28. In brief, splenocytes were prepared by passing spleen through a cell strainer diluted in sterile PBS (7.4 pH). The single-cell suspensions of peripheral blood mononuclear cells (PBMCs) were collected and added onto an equal volume of Ficoll-paque plus solution (Fisher Scientific, MA). Red blood cells were removed by centrifugation at 450 × g for 30 minutes at 4°C. The splenocytes in the interface were harvested and cells were plated at 5 × 10^5^ cells per well, as duplicates per sample, in 100 μL of RPMI-1640 (Sigma Aldrich, MO) supplemented with 10% fetal bovine serum (FBS) and 1% Penicillin and Streptomycin. Cells were stimulated with either 0.05 mg/mL, 0.1 mg/mL, 0.20 mg/mL OMPs or 0.05 mg/mL, 0.1 mg/mL flagellin proteins, and incubated for 3 days at 37 °C in the presence of 5% CO_2_. As a positive control, splenocytes stimulated with 0.0 mg/mL of antigen were used. The PBMC proliferation was measured using 3-(4,5-dimethylthiazol-2-yl)-2,5-diphenyltetrazolium bromide (MTT) assay as previously described [18]. The optical density was measured at 570 nm using a spectrophotometer as described above.

#### *Salmonella* loads in the ceca of vaccinated birds

Ceca samples were collected from one bird/pen (n = 6) at d28 and analyzed for *S*. Enteritidis loads by plating. Ceca samples were collected into stomacher bags, placed on ice, and transported to the laboratory. The ceca samples were enriched with 3× (wt/vol) buffered peptone water (BPW) (Thermo Fisher Scientific, MA), macerated using a rubber mallet, and then stomached for 1 minute and incubated for 12 hours at 41°C. A volume of 100 μL of ceca was serially diluted into 900 μL of BPW and from every dilution, a volume of 10 μL was plated in duplicates on Xylose Lactose Tergitol^™^ 4 (XLT4) (Neogen, MI) agar plates. Plates were then incubated for 24 hours at 41°C for confirmation of black colonies. *Salmonella* enumeration data were recorded as CFU/g of ceca and then transformed to Log 10 CFU/g of ceca for statistical analysis.

#### IL-1β, IFN-γ, IL-10 and iNOS gene expression in the cecal tonsils of vaccinated birds

Cecal tonsil samples were collected from one bird/pen (n = 6) at d28 of age. For gene expression analysis, all treatment groups consisted of six samples, in duplicates. Total RNA was extracted using TRIzol reagent (Invitrogen, CA) per the manufacturer’s instructions. The isolated RNA was dissolved in Tris-EDTA Buffer (pH 7.5), and the purity of the RNA was determined by measuring absorbance in a NanoDrop spectrophotometer (NanoDrop^™^ 2000c). The cDNA synthesis was performed with 2 μg of total RNA template in a 20μl reaction volume (reaction buffer, 0.1M DTT, 10mM dNTPs, 1 μmol of oligo (dT) primer, 10 units of RNAsin and 100 units of M-MLV reverse transcriptase (Promega, WI) at 40°C for 1 hour and followed by 95°C for 10 minutes. The mRNA transcripts were analyzed for IL-1β, IFN-γ, IL-10, and iNOS by RT-PCR (CFX96 Touch Real-Time System, BioRad) using iQ^™^ SYBR^®^ Green Supermix (ThermoFisher Scientific, MA). The reaction mixture consisted of 5μl name of PowerTrack^™^ SYBR Green Master Mix (Thermo Fisher Scientific, MA), 1μl of cDNA (0.1 ng) template, 20μM of each forward and reverse primer, and made up to 10μl with RNAse-free H_2_O. The amplification protocol includes: an initial denaturation at 95°C for 5 minutes (1 cycle), followed by 95°C for 10 seconds, and 60.0°C for 45 seconds (40 cycles). The specificity of the of the RT-PCR product was verified through the melting curve generation at the end of each run. The housekeeping gene β-actin was used as a reference gene for the normalization of the Ct values. Fold change from the reference was calculated, as explained previously [19]. In brief, all data were normalized to the mRNA level of the control group and were reported as the fold-change (2^−ΔΔ^Ct method). The primers sequences used for RT-PCR analysis in this study are described in Table 2.

**Table 2:** Real-time quantitative RT-PCR primers.

Target Gene	Sequence	T_a_	Reference
IL-1β (F)	5′-TCCTCCAGCCAGAAAGTGA-3′	57.0 °C	[20]
IL-1β (R)	5′-CAGGCGGTAGAAGATGAAGC-3′		
IFN-γ (F)	5’-GTGAAGAAGGTGAAAGTATCATGGA-3’	57.0 °C	[21]
IFN-γ (R)	5’-GCTTTGCGCTGGATTCTCA-3’		
IL-10 (F)	5′-CATGCTGCTGGGCCTGAA-3′	57.5 °C	[22]
IL-10 (R)	3′ -CGTCTCCTTGATCTGCTTGATG-5′		
iNOS (F)	5′-AGTGGTATGCTCTGCCTGCT-3′	60.0 °C	[23]
iNOS (R)	3′ -CCAGTCCCATTCTTCTTCC-5′		
β-actin (F)	5′-ACCGGACTGTTACCAACACC-3′	57.0 °C	[24]
β-actin (R)	3′ -GACTGCTGCTGACACCTTCA-5′		

Abbreviations: F, forward; R, reverse; T_a_, annealing temperature

### Statistical analysis

For this experiment, the experimental unit was the pen, n = 6 pen/treatment. Before vaccination, all birds were randomly assigned treatment groups. After vaccination, each pen was randomly assigned 7 technical replicates as birds/pen. Serum, cloacal swabs, bile, spleen, cecal tonsils, and ceca samples were taken from 1 bird/pen at each time point, and samples were analyzed in duplicates. When data were normally distributed the difference between the four experimental treatments were determined using a One-way analysis of variance (ANOVA), followed by Tukey’s post hoc test for multiple comparisons. Otherwise, the statistical differences between the four experimental treatments were determined using a Kruskal-Wallis Test, followed by Dunn post-hoc test for multiple comparisons. All statistical analyzes were performed using JMP Pro 14 (SAS Institute Inc., USA). The results were classified as statistically significant at P<0.05.

## Results

### The effects of *Salmonella* CNP vaccine on production performance of vaccinated birds

The administration of the CNP vaccine did not significantly affect the production performance of the immunized birds. There were no significant differences (P>0.05) in the mean BWG or FCR of birds in any of the treatment groups at all time points, compared to control. The final BWG and FCR at d28 of age is reported in Table 3.

**Table 3:** The effects of *Salmonella* CNP vaccine on production performance of vaccinated birds.

Treatment	d0 to d28	
	BWG (g)	FCR
**No vaccine**	1439	2.03
**Live vaccine**	1448	1.78
**Killed vaccine**	1458	1.82
**Live+killed vaccine**	1423	1.95
**SEM**	16	0.06
**P-value**	0.91	0.44

The *Salmonella* CNP vaccine was synthesized with *S*. Enteritidis OMPs and flagellin proteins. At d1 of age birds were allocated into treatment groups: 1) No vaccine, 2) Live vaccine, 3) CNP vaccine, or 4) Live+CNP vaccine. At d1 of age, birds were orally vaccinated with PBS, Live vaccine, or CNP. At d7 of age, the No vaccine, Live vaccine and CNP vaccine groups were boosted with PBS and the Live+CNP vaccine group was boosted with CNP. At d14 of age, birds were orally challenged with 1 × 10^9^ CFU/bird of *S*. Enteritidis. Production performance of the birds was monitored weekly. All birds were euthanized at 4 wk of age (d28). Data represents 6 pens/treatment. Significant differences between the groups were determined by one-way ANOVA, followed by Tukey post-hoc test. Differences (±SEM) were considered statistically significant at P<0.05. No vaccine: mock PBS; Live vaccine: POULVAC^®^ ST vaccine; CNP vaccine: 10μg killed CNP vaccine; Live+CNP vaccine: POULVAC^®^ ST vaccine + 10μg killed CNP vaccine booster.

### The effects of *Salmonella* CNP vaccine on anti-OMPs specific IgY and IgA antibodies of vaccinated birds

#### A. Antigen-specific IgY in serum samples

**Fig 1:**
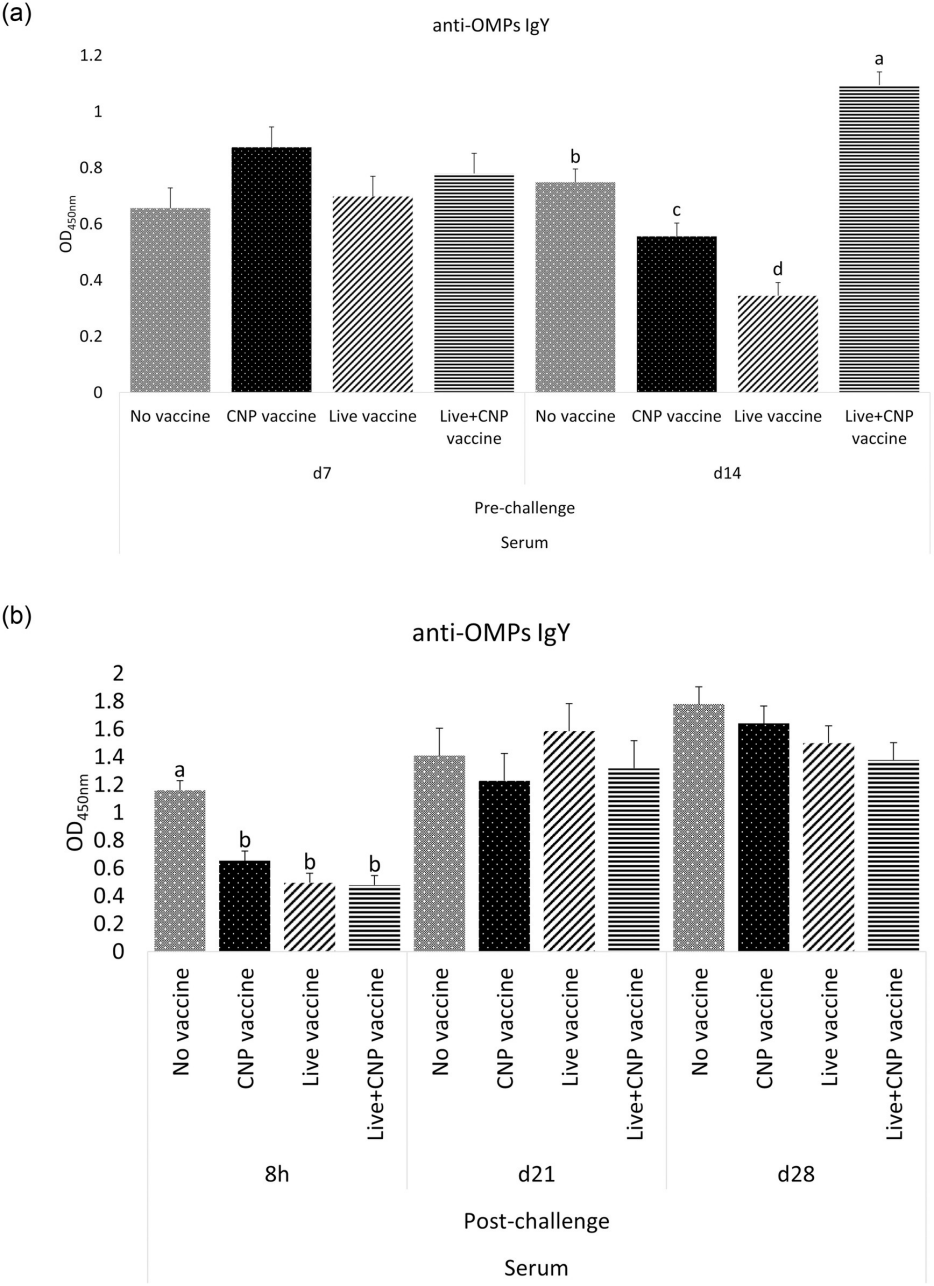
Effects of *Salmonella* CNP vaccine on anti-OMPs specific IgY antibodies of vaccinated birds. At d1 of age birds were allocated into treatment groups: 1) No vaccine, 2) Live vaccine, 3) CNP vaccine, or 4) Live+CNP vaccine. At d1 of age, birds were orally vaccinated with PBS, Live vaccine, or CNP. At d7 of age, the No vaccine, Live vaccine and CNP vaccine groups were boosted with PBS and the Live+CNP vaccine group was boosted with CNP. At d14 of age, birds were orally challenged with 1 × 10^9^ CFU/bird of *S*. Enteritidis. Blood samples were collected pre- and post-challenge and analyzed for anti-*Salmonella* OMPs-specific IgY levels by ELISA (n = 6). (A) Pre-challenge; (B) Post-challenge. Results were reported as average optical density (OD) values. Significant differences between the groups were determined by Kruskal-Wallis Test, followed by Dunn post-hoc test. Bars (+SE) with no common superscript differ (P<0.05). No vaccine: mock PBS; Live vaccine: POULVAC^®^ ST vaccine; CNP vaccine: 10μg killed CNP vaccine; Live+CNP vaccine: POULVAC^®^ ST vaccine + 10μg killed CNP vaccine booster.

At d7 of age, birds in all treatment groups had no significant differences (P>0.05) of anti-*Salmonella* OMPs-specific IgY in serum, compared to control (Fig 1A). At d14 of age, Live+CNP-vaccinated birds had 46% greater levels (P<0.05) of anti-*Salmonella* OMPs IgY in serum, compared to control (Fig 1A). At d14 of age, CNP and Live-vaccinated birds had 19% and 40% lower levels (P<0.05) of anti-*Salmonella* OMPs IgY in serum, compared to control (Fig 1A). At 8h post-challenge, CNP, Live, and Live+CNP-vaccinated birds had 44%, 57%, and 59% lower levels (P<0.05) of anti-*Salmonella* OMPs IgY in serum, respectively, compared to control (Fig 1B). At d21 of age, birds in all treatment groups had no significant differences (P>0.05) of anti-*Salmonella* OMPs-specific IgY in serum, compared to control (Fig 1B). At d28 of age, birds in all treatment groups had no significant differences (P>0.05) of anti-*Salmonella* OMPs-specific IgY in serum, compared to control (Fig 1B).

#### B. Antigen-specific IgA in cloacal swab samples

**Fig 2:**
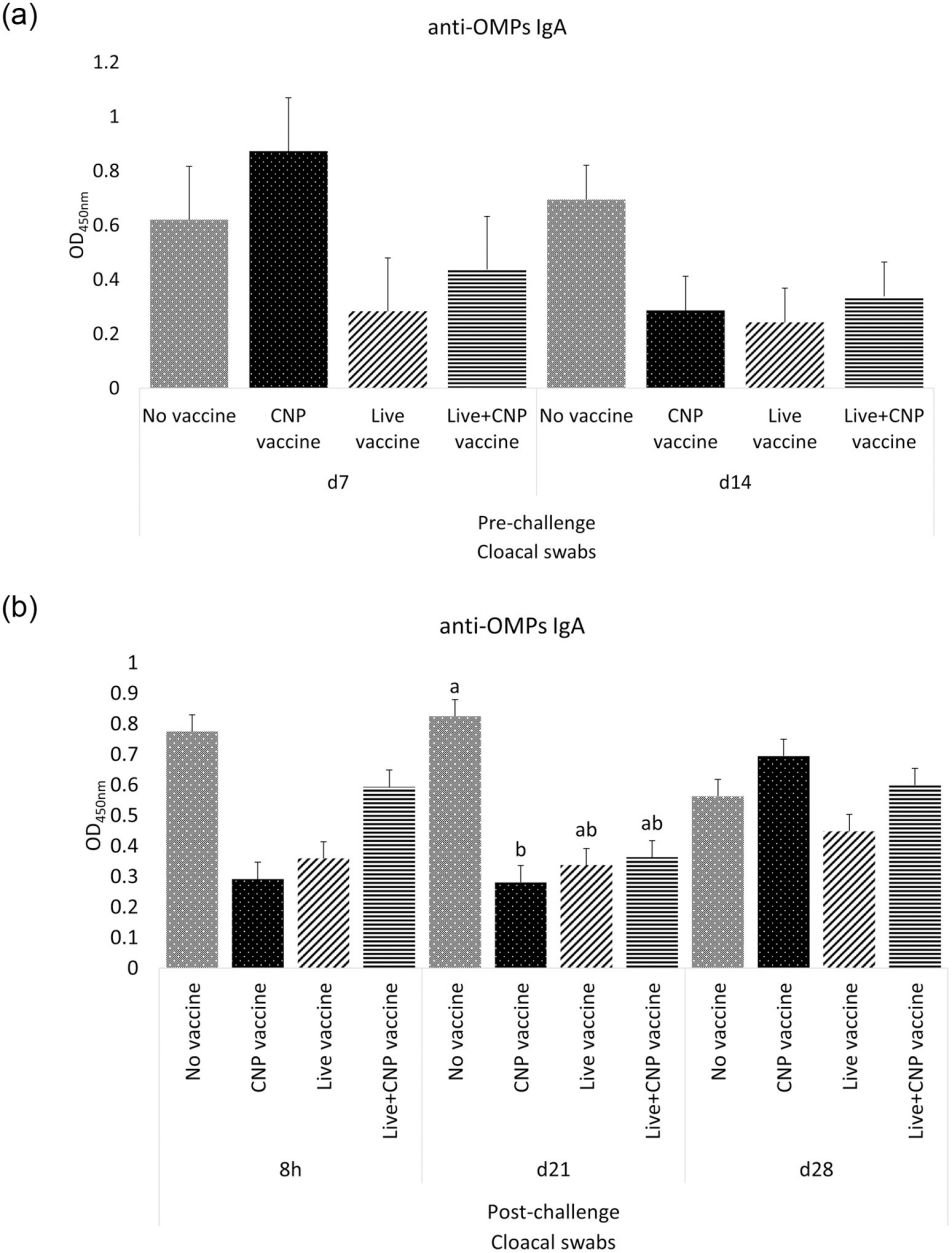
Effects of *Salmonella* CNP vaccine on anti-OMPs specific IgA antibodies of vaccinated birds. At d1 of age birds were allocated into treatment groups: 1) No vaccine, 2) Live vaccine, 3) CNP vaccine, or 4) Live+CNP vaccine. At d1 of age, birds were orally vaccinated with PBS, Live vaccine, or CNP. At d7 of age, the No vaccine, Live vaccine and CNP vaccine groups were boosted with PBS and the Live+CNP vaccine group was boosted with CNP. At d14 of age, birds were orally challenged with 1 × 10^9^ CFU/bird of *S*. Enteritidis. Cloacal swabs samples were collected pre- and post-challenge and analyzed for anti-*Salmonella* OMPs-specific IgA levels by ELISA (n = 6). (A) Pre-challenge; (B) Post-challenge. Results were reported as average optical density (OD) values. Significant differences between the groups were determined by Kruskal-Wallis Test, followed by Dunn post-hoc test. Bars (+SE) with no common superscript differ (P<0.05). No vaccine: mock PBS; Live vaccine: POULVAC^®^ ST vaccine; CNP vaccine: 10μg killed CNP vaccine; Live+CNP vaccine: POULVAC^®^ ST vaccine + 10μg killed CNP vaccine booster.

At d7 of age, birds in all treatment groups had no significant differences (P>0.05) of anti-*Salmonella* OMPs-specific IgA in cloacal swabs, compared to control (Fig 2A). At d14 of age, birds in all treatment groups had no significant differences (P>0.05) of anti-*Salmonella* OMPs-specific IgA in cloacal swabs, compared to control (Fig 2A). At 8h post-challenge, birds in all treatment groups had no significant differences (P>0.05) of anti-*Salmonella* OMPs-specific IgA in cloacal swabs, compared to control (Fig 2B). At d21 of age, CNP, Live, and Live+CNP-vaccinated birds had 66%, 59%, and 56% lower levels (P<0.05) of anti-*Salmonella* OMPs IgA in cloacal swabs, respectively, compared to control (Fig 2B). At d28 of age, birds in all treatment groups had no significant differences (P>0.05) of anti-*Salmonella* OMPs-specific IgA in cloacal swabs (Fig 2B).

#### C. Antigen-specific IgA in bile samples

**Fig 3:**
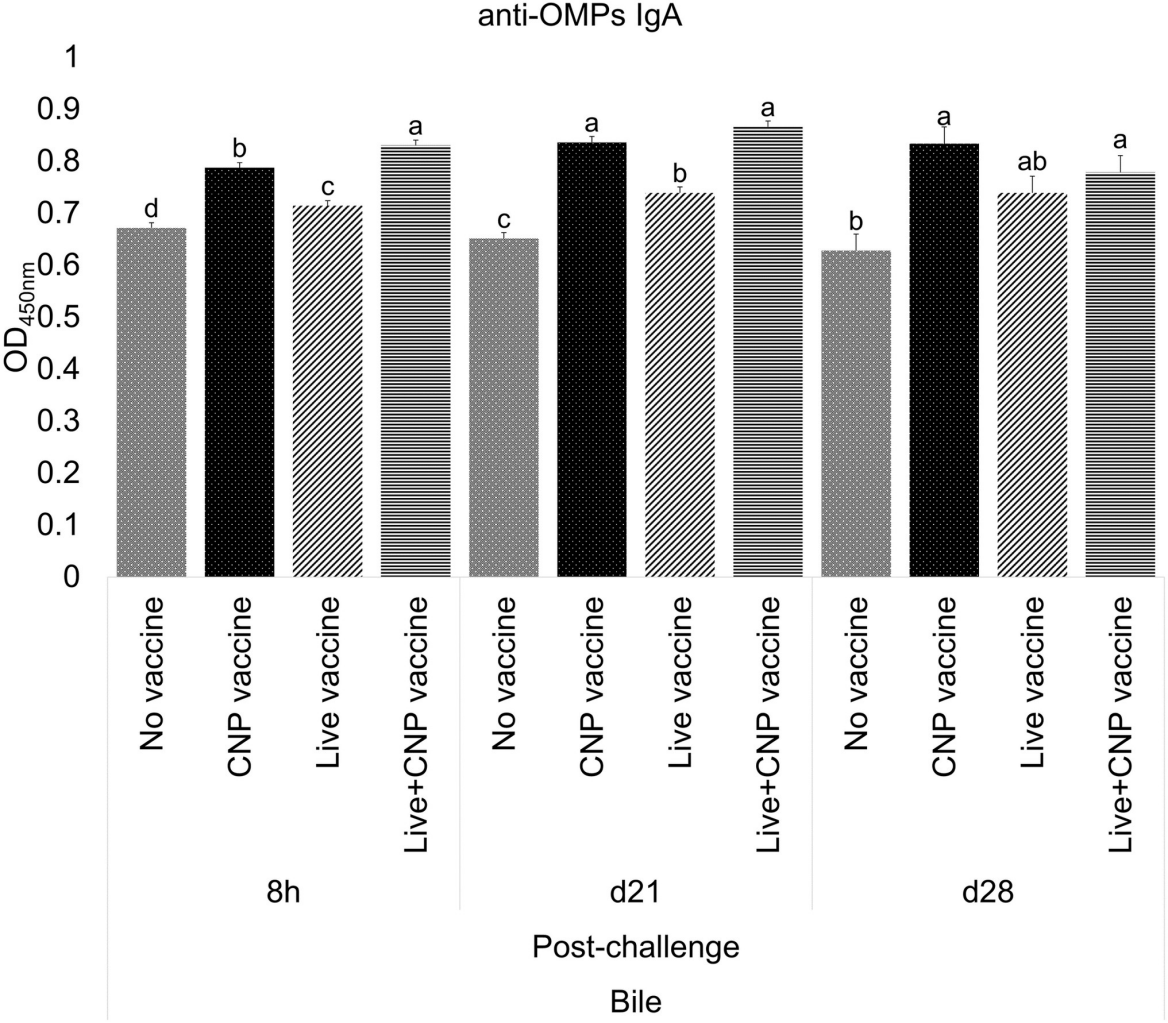
Effects of *Salmonella* CNP vaccine on anti-OMPs specific IgA antibodies of vaccinated birds. At d1 of age birds were allocated into treatment groups: 1) No vaccine, 2) Live vaccine, 3) CNP vaccine, or 4) Live+CNP vaccine. At d1 of age, birds were orally vaccinated with PBS, Live vaccine, or CNP. At d7 of age, the No vaccine, Live vaccine and CNP vaccine groups were boosted with PBS and the Live+CNP vaccine group was boosted with CNP. At d14 of age, birds were orally challenged with 1 × 10^9^ CFU/bird of *S*. Enteritidis. Bile samples were collected post-challenge and analyzed for anti-*Salmonella* OMPs-specific IgA levels by ELISA (n = 6). Results were reported as average optical density (OD) values. Significant differences between the groups were determined by Kruskal-Wallis Test, followed by Dunn post-hoc test. Bars (+SE) with no common superscript differ (P<0.05). No vaccine: mock PBS; Live vaccine: POULVAC^®^ ST vaccine; CNP vaccine: 10μg killed CNP vaccine; Live+CNP vaccine: POULVAC^®^ ST vaccine + 10μg killed CNP vaccine booster.

At 8h post-challenge, CNP and Live+CNP-vaccinated birds had 17% and 24% greater levels (P<0.05) of anti-*Salmonella* OMPs IgA in bile, respectively, compared to control (Fig 3). At d21 of age, CNP and Live+CNP-vaccinated birds had 17% and 24% greater levels (P<0.05) of anti-*Salmonella* OMPs IgA in bile, respectively, compared to control (Fig 3). At d28 of age, CNP, Live, and Live+CNP-vaccinated birds had 33%, 18%, and 24% greater levels (P<0.05) of anti-*Salmonella* OMPs IgA in bile, respectively, compared to control (Fig 3).

### The effects of *Salmonella* CNP vaccine on antigen recall response of vaccinated birds

**Fig 4:**
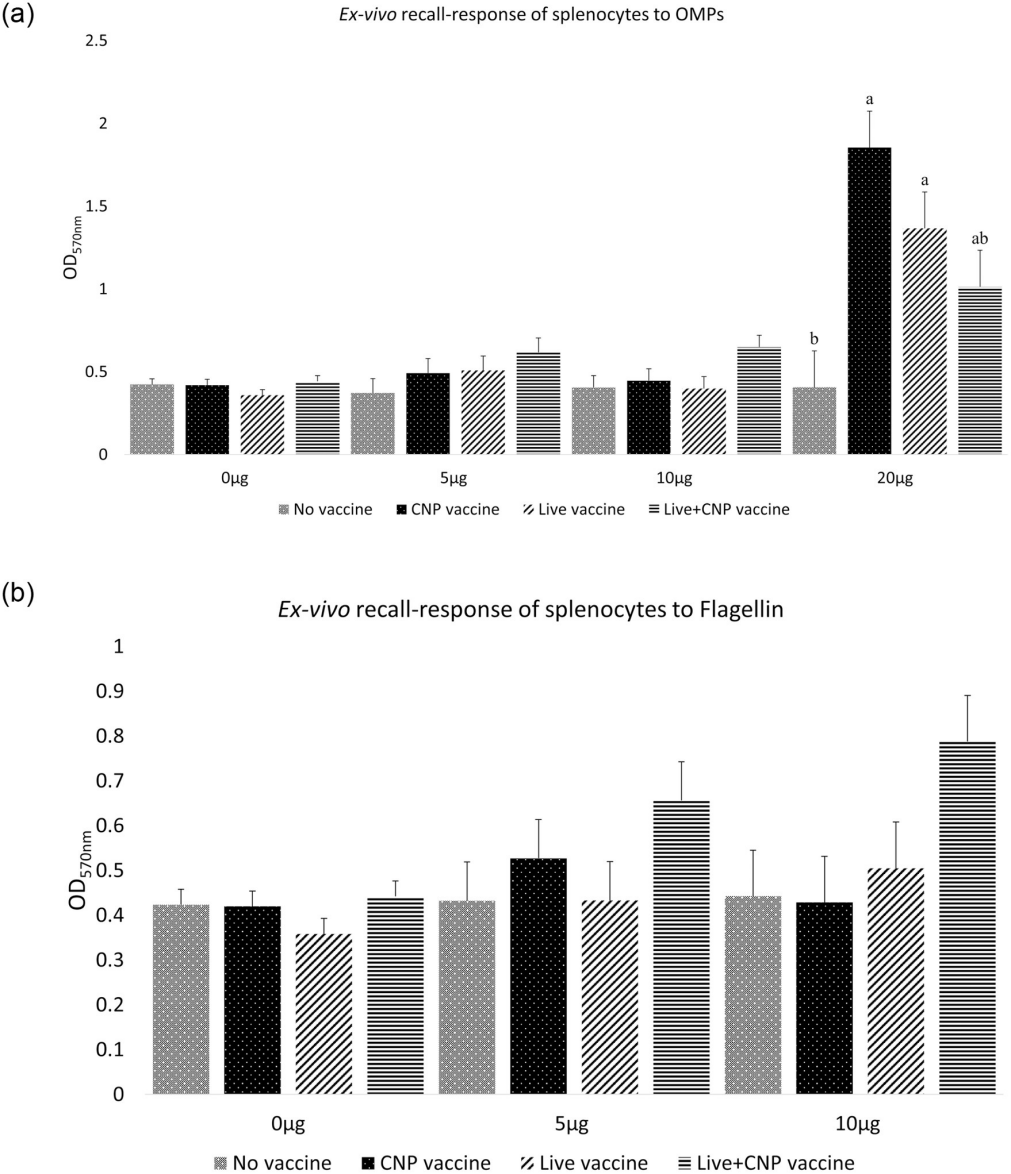
Ex-vivo recall-response of splenic PBMCs of vaccinated birds. At d1 of age birds were allocated into treatment groups: 1) No vaccine, 2) Live vaccine, 3) CNP vaccine, or 4) Live+CNP vaccine. At d1 of age, birds were orally vaccinated with PBS, Live vaccine, or CNP. At d7 of age, the No vaccine, Live vaccine and CNP vaccine groups were boosted with PBS and the Live+CNP vaccine group was boosted with CNP. At d14 of age, birds were orally challenged with 1 × 10^9^ CFU/bird of *S*. Enteritidis. Splenocytes PBMCs of broilers at d21 of age were harvested and stimulated with either 0.05 mg/mL, 0.1 mg/mL, 0.20 mg/mL OMPs or 0.05 mg/mL, 0.1 mg/mL flagellin proteins for 3 days. As a positive control, splenocytes stimulated with 0.0 mg/mL of antigen were used. Recall response was measured using MTT assay and absorbance was measured at OD570nm. (A) OMPs; (B) Flagellin. Results were reported as average optical density (OD) values. Significant differences between the groups were determined by one-way ANOVA, followed by Tukey post-hoc test. Bars (+SE) with no common superscript differ (P<0.05). No vaccine: mock PBS; Live vaccine: POULVAC^®^ ST vaccine; CNP vaccine: 10μg killed CNP vaccine; Live+CNP vaccine: POULVAC^®^ ST vaccine + 10μg killed CNP vaccine booster.

An *ex-vivo* antigen recall response assay was performed to analyze the OMPs antigen-specific lymphocyte proliferative response of immunized birds. At d21 of age, PBMCs from birds in the CNP vaccine and Live vaccine groups had a significant increase (P<0.05) in T-lymphocyte proliferation at 0.02 mg/mL OMPs stimulation compared to the control group (Fig 4A).

An *ex-vivo* antigen recall response assay was performed to analyze the Flagellin antigen-specific lymphocyte proliferative response of immunized birds. At d21 of age, there were no significant differences in T-lymphocyte proliferation, in response to 0.0 mg/mL, 0.05 mg/mL, or 0.1 mg/mL flagellin stimulation, between all vaccinated groups compared to control (Fig 4B).

### The effects of *Salmonella* CNP vaccine on *Salmonella* load in the ceca of vaccinated birds

**Fig 5:**
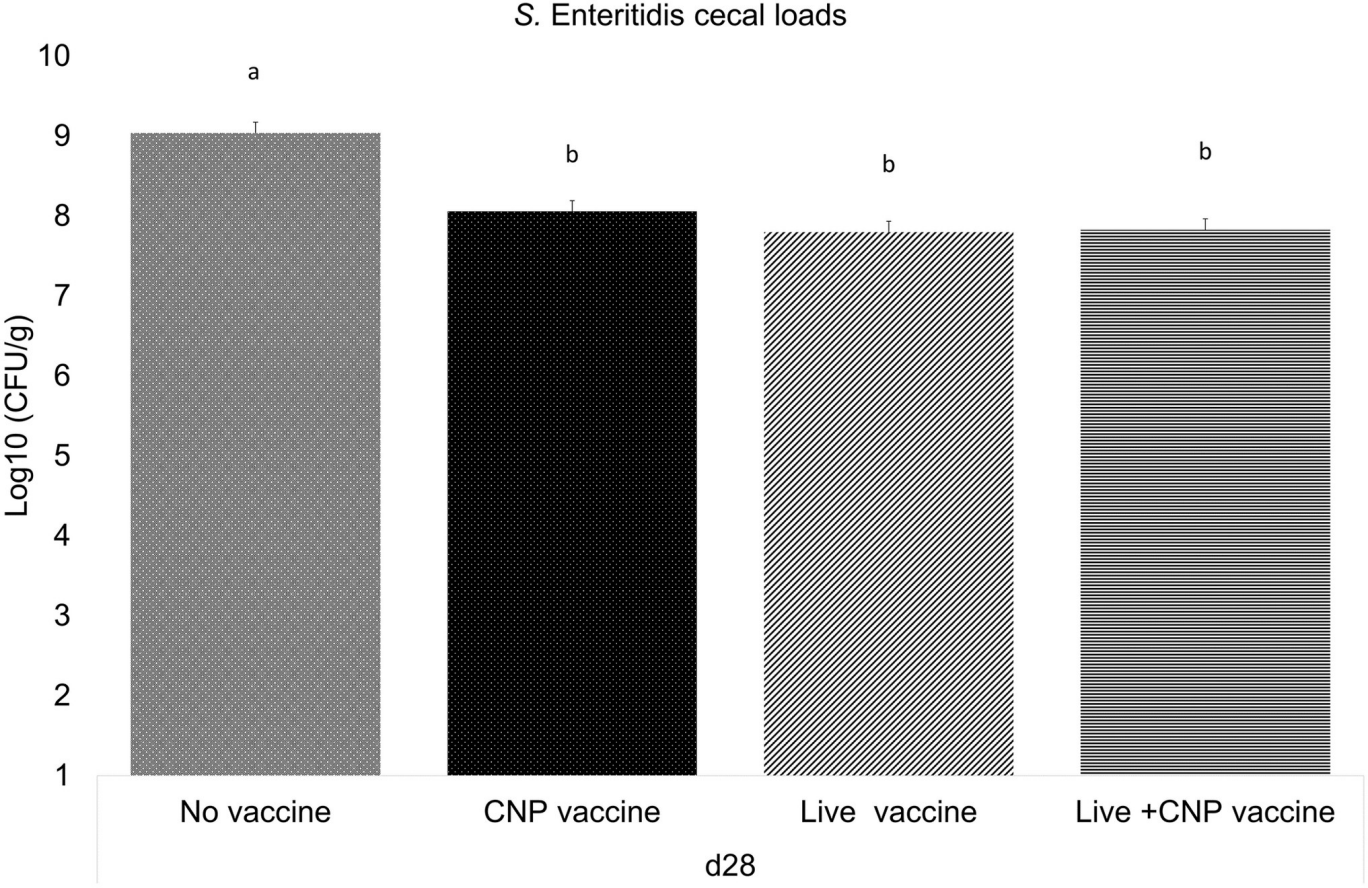
*Salmonella* loads in the ceca of vaccinated birds. At d1 of age birds were allocated into treatment groups: 1) No vaccine, 2) Live vaccine, 3) CNP vaccine, or 4) Live+CNP vaccine. At d1 of age, birds were orally vaccinated with PBS, Live vaccine, or CNP. At d7 of age, the No vaccine, Live vaccine and CNP vaccine groups were boosted with PBS and the Live+CNP vaccine group was boosted with CNP. At d14 of age, birds were orally challenged with 1 × 10^9^ CFU/bird of *S*. Enteritidis. Ceca samples were collected from 1 birds/pen, stomached, and enriched for enumeration by plating on XLT-4 agar. *Salmonella* enumeration data were recorded as CFU/g of ceca and then transformed to Log 10 CFU/g of ceca for statistical analysis. Significant differences between the groups were determined by one-way ANOVA, followed by Tukey post-hoc test. Bars (+SE) with no common superscript differ (P<0.05). No vaccine: mock PBS; Live vaccine: POULVAC^®^ST vaccine; CNP vaccine: 10μg killed CNP vaccine; Live+CNP vaccine: POULVAC^®^ ST vaccine + 10μg killed CNP vaccine booster.

The *S*. Enteritidis loads in the ceca were monitored for all treatment groups. At d28 of age, CNP and Live+CNP-vaccinated groups had 0.9 Log10 CFU/g and 1 Log10 CFU/g decreased *S*. Enteritidis ceca load (P<0.05), respectively, compared to control (Fig 5).

### The effects of *Salmonella* CNP vaccine on IL-1β, IFN-γ, IL-10 and iNOS gene expression in the ceca of vaccinated birds

**Fig 6:**
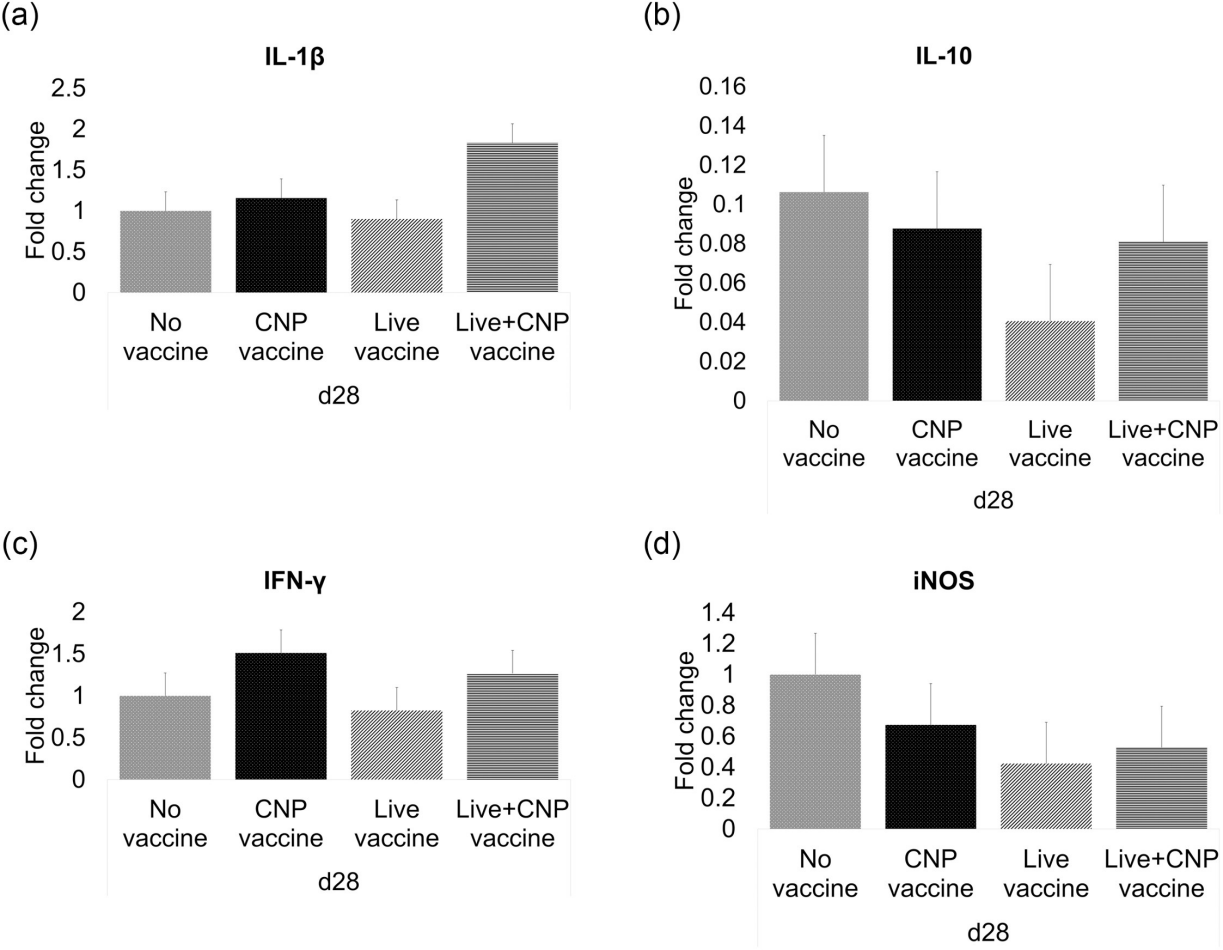
Cytokine gene expression in the cecal tonsils of vaccinated birds. At d1 of age birds were allocated into treatment groups: 1) No vaccine, 2) Live vaccine, 3) CNP vaccine, or 4) Live+CNP vaccine. At d1 of age, birds were orally vaccinated with PBS, Live vaccine, or CNP. At d7 of age, the No vaccine, Live vaccine and CNP vaccine groups were boosted with PBS and the Live+CNP vaccine group was boosted with CNP. At d14 of age, birds were orally challenged with 1 × 10^9^ CFU/bird of *S*. Enteritidis. Cecal tonsil samples were collected at d28 and analyzed for cytokine mRNA amounts by RT-PCR. Data represented fold change compared to control. (A) IL-1β mRNA; (B) IL-10 mRNA; (C) IFN-γ; (D) iNOS mRNA. Significant differences between the groups were determined by one-way ANOVA, followed by Tukey post-hoc test. Bars (+SE) with no common superscript differ (P<0.05). No vaccine: mock PBS; Live vaccine: POULVAC^®^ST vaccine; CNP vaccine: 10μg killed CNP vaccine; Live+CNP vaccine: POULVAC^®^ ST vaccine + 10μg killed CNP vaccine booster.

The extracted total RNA from the cecal tonsils of immunized birds were analyzed for the expression of IL-1β, IFN-γ, IL-10 and iNOS mRNA by RT-PCR. At d28 of age, there were no significant differences in IL-1β (Fig 6A), IL-10 (Fig 6B), IFN-γ (Fig 6C), and iNOS (Fig 6D) mRNA between vaccinated groups compared to control.

## Discussion

The oral *Salmonella* CNP vaccine for poultry has been previously characterized by our collaborators and has shown to be a viable alternative to current vaccines [9, 25]. *Salmonella* CNP vaccine was synthesized with *S*. Enteritidis OMPs and flagellin proteins and surface coated with flagellin. Previous studies have identified that CNPs are biocompatible in chickens [9]. *In-vitro* and *in-vivo* studies have shown that loaded CNP vaccine against *Salmonella* has a high cationic charge and spherical shape with an average particle size distribution of 500 nm, which facilitates their delivery and adhesion to the anionic mucosal layer of the gut and their uptake by M-cells in the Peyer’s Patches [9]. Other studies have shown that the oral CNP vaccine for *Salmonella* upregulates toll-like receptor and Th1 and Th2 cytokines mRNA expression as well as enhance antigen-specific systemic and mucosal antibody response, and reduce the *Salmonella* load in the ceca [14, 17, 25]. Previous studies have also shown that empty CNPs alone do not improve antibody response in the absence of a *Salmonella* antigen [9]. The scope of this study was to examine an alternative vaccination scheme for chickens by testing the efficacy of administering a live *Salmonella* vaccine followed by a killed *Salmonella* CNP vaccine booster on broilers.

In this study, we monitored the bird’s BWG and FCR weekly. Results show that the *Salmonella* CNP vaccine had no significant effects on the production performance parameters of vaccinated birds. One of the predominant desirable features of an ideal vaccine includes safety. Hence, non-significant BWG and FCR results can be seen as positive results because it shows that the vaccine treatment does not impact production performance. Our findings are in agreement with previous studies that have shown that CNP vaccines are safe to use in broilers and layers [9, 14, 17, 18, 26]. Results indicate that the oral *Salmonella* CNP vaccine is a safe vaccine candidate that does not negatively affect the production performance of chickens.

This study quantified the anti-*Salmonella* OMPs IgY levels in serum and the anti-*Salmonella* OMPs IgA levels in cloacal swabs and bile samples from vaccinated birds. Polymeric IgA is the most abundant antibody in the mucosal immune system [27], and it is considered the first line of defense for enteric pathogens. Orally delivered vaccines are appropriate to protect the host against pathogenic bacteria that invade the intestinal mucosa as they mimic the natural route infection of many enteric pathogens. Injected vaccines induce a lower mucosal immunity [28], whereas a successful oral vaccine induces significantly increasing IgA levels against the pathogen of interest. The administration of *Salmonella* CNP vaccine and the Live+CNP vaccine induced significantly higher levels of antigen-specific IgA in bile samples of vaccinated birds, in response to bacterial challenge, compared to control. The levels of mucosal IgA antibody response in bile samples were consistent with other studies using the vaccine under study in layers and broilers [9, 14, 25]. This consistent increasing mucosal antibody response can be attributed to the experimental design of the vaccine, which can thrive in acidic environments, its high cationic charge ensures delivery of the antigen to the intestinal anionic mucous layers, the major immunodominant proteins of *S*. Enteritidis, the surface-tagged flagellin proteins facilitate antigen recognition by TLR 5 [5], and the average size of 500 nm facilitates the cell uptake for antigen presentation and development of memory T-cells.

Various studies have shown that IgG can also contribute to mucosal immune defense [29, 30]. For systemic IgY response, findings show that the combination of the Live+CNP vaccine substantially increased the antigen-specific IgY levels at d14 of age. Results agree with other studies that show that the CNP vaccine can also induce substantial levels of antigen-specific systemic IgY [14]. It may be that the observed elevated levels of IgY may represent an enhanced humoral immune response due to a synergistic effect of the Live+CNP vaccine combination. However, it is unclear why the administration of the CNP and Live vaccine alone resulted in a significant decrease of antigen-specific IgY levels at d14 of age when compared to the control. Nonetheless, for mucosal pathogens the production of IgA is more important because injected vaccines fail to provide protection at mucosal sites [31] where pathogens, like *Salmonella*, make their entry and persist. It is also known that mucosal vaccination generates an efficient mucosal immunity, but may not always induce a strong systemic immune response [31]. At 8h post-challenge, the non-vaccinated control group had significantly higher antigen-specific IgY levels when compared to other treatment groups. This is consistent with other studied where increasing systemic IgY antibody levels can be attributed to a persistent infection in carrier animals [14, 32]. Our findings demonstrate that the administration of the CNP vaccine, either as a first dose or as a booster vaccination, can elicit an OMPs-specific humoral immune response.

For this study, we quantified the antigen-recall response by antigen-specific lymphocyte proliferation assay to measure the cell-mediated immune response of vaccinated birds against *Salmonella*. The goal of vaccination is to elicit an immune response that creates immunological memory to a specific antigen. The rapid recall response upon re-exposure to an antigen is critical in preventing infection and also in controlling the extent of the infection [33]. Findings demonstrate that the immunogenic *Salmonella* OMPs can induce significantly higher lymphocyte proliferation in chickens [9, 34]. The observed vaccine-induced recall response post-vaccination is in agreement with previous studies of PBMCs recall response against loaded CNPs antigen stimulation [9]. Our results indicate that 0.02 mg of loaded OMPs into CNP can induce a significant antigen-specific proliferation upon re-exposure to *Salmonella*. Also, the recall response to *Salmonella* OMPs antigens may be dependent on antigen type and stimulated concentration as no significant antigen recall response was observed with flagellin stimulation and the antigen proliferation was substantially greater with increasing concentrations of OMPs. Future studies will take into account the vaccination with 0.02 mg CNP loaded antigen for a better memory recall response of immunized birds.

In this study, we quantified the *S*. Enteritidis loads in the ceca of vaccinated birds. As broiler growth rate has improved throughout the years with genetic selection, modern day birds can reach slaughter weight as early as between 4 and 7 weeks of age [35, 36]. For this reason, the *Salmonella* cecal loads were monitored at 4 weeks of age; at a 2wk post-challenge period. All treatment groups were kept under the same experimental conditions; hence, all birds were challenged with *S*. Enteritidis. The reduction of *Salmonella* loads in poultry is critical to ultimately decrease foodborne illness risks for humans. Chickens can live with up to Log 5 *Salmonella* CFU and remain asymptomatic [3], but the infective dose can be as low as Log 3 *Salmonella* CFU for individuals that use antacids [37]. For chickens, the resistance to *Salmonella* is attributed to the upregulation of chicken T-regulatory (T-reg) cells and the downstream suppressive immune response by IL-10 [38]. Results show that at 2wk post-challenge the *S*. Enteritidis loads in the ceca of birds immunized with CNP vaccine or with Live+CNP vaccine was significantly reduced compared to the non-vaccinated control. A 1 Log reduction of *Salmonella* in the ceca of broilers is of biological importance because in combination with active “on-farm” and processing interventions [39–41], it may result in fewer contaminated carcasses that may lead to a significant reduction in the incidence of salmonellosis in humans. For this reason, future research will study the impact of CNP vaccination on broiler carcass loads. Overall, findings demonstrate that the administration of the *Salmonella* CNP vaccine, either alone or as a booster vaccination, is a viable prevention method against *Salmonella*.

Interleukin-1β is a key cytokine that mediates the inflammatory responses to infections [5], while IL-10 is an anti-inflammatory cytokine that has been linked to *Salmonella* resistance in broilers [38]. The CNP vaccination had no significant effects on the pro-inflammatory cytokine IL-1β mRNA expression levels or the anti-inflammatory cytokine IL-10 mRNA expression levels in the cecal tonsils of birds at the end of the experimental period. However, there was an increase in IL-1β mRNA expression in cecal tonsils of birds immunized with CNP vaccine and birds vaccinated with Live+CNP vaccine, compared to control. This is in agreement with the previous studies with CNP vaccine using layers and broilers [9, 14]. The oral delivery of the *Salmonella* CNP vaccine has been shown to induce predominantly a Th1-type inflammatory response that has been attributed to the intrinsic adjuvant composition of the vaccine [42]. Results also show that the IL-10 cytokine levels were increased in the non-vaccinated control group which can be attributed to the concurrent *Salmonella* resistance seen in poultry. Overall, the administration of the CNP vaccine had no negative effects on the IL-1β or IL-10 cytokine mRNA levels of birds at d28 of age.

Interferon-gamma is a critical pro-inflammatory cytokine that can promote macrophage activation [5] and iNOS is a key enzyme in the macrophage inflammatory response that induces the production of nitric oxide (NO) to eliminate pathogens like *Salmonella* [43]. Both IFN-γ and iNOS are critical during infections by intracellular pathogens. However, when macrophages are activated during infections, NO can be produced at high levels that can result in toxic reactions against the hosts’ tissues [44] and could compromise the production performance of broilers. For this reason, the absence of significance for IFN-γ and iNOS mRNA expression levels at d28 of age can be seen as an indication that the birds’ production performance is not adversely compromised by the CNP vaccination.

## Conclusion

We studied an alternative vaccination scheme for chickens by testing the efficacy of administering a live *Salmonella* vaccine followed by a killed *Salmonella* CNP vaccine booster on broilers. In sumamry, the single or combined administration of the *Salmonella* CNP vaccine to broiler birds can 1) induce an antigen-specific immune response against *S*. Enteritidis, 2) can activate the intestinal mucosal immune system, 3) can decrease the *S*. Enteritidis loads in ceca, and 4) does not have adverse effects on the bird’s IL-1β, IFN-γ, IL-10 or iNOS mRNA expression levels or production performance parameters at d28 of age. The results of the present study showed that the CNP vaccine can be administered either as a first dose or as a booster vaccination, making it an alternative vaccine candidate against *S*. Enteritidis in broilers. Future studies will further examine the vaccine’s potential for mass vaccination methods in the poultry industry.

## Abbreviations

BWG: Body weight gain
CNP: Chitosan nanoparticle
FCR: Feed conversion ratio
OMPs: Outer membrane proteins
*S*. Enteritidis: *Salmonella* Enteritidis
